# Relative sea‐level change regulates organic carbon accumulation in coastal habitats

**DOI:** 10.1111/gcb.14558

**Published:** 2019-01-24

**Authors:** Kenta Watanabe, Koji Seike, Rumiko Kajihara, Shigeru Montani, Tomohiro Kuwae

**Affiliations:** ^1^ Coastal and Estuarine Environment Research Group Port and Airport Research Institute Yokosuka Japan; ^2^ Geological Survey of Japan National Institute of Advanced Industrial Science and Technology (AIST) Tsukuba Japan; ^3^ Civil Engineering Research Institute for Cold Region Sapporo Japan; ^4^ Graduate School of Environmental Science Hokkaido University Sapporo Japan

**Keywords:** climate change, coastal habitats, land subsidence, organic carbon accumulation, relative sea‐level change, saltmarshes, seagrass meadows

## Abstract

Because coastal habitats store large amounts of organic carbon (C_org_), the conservation and restoration of these habitats are considered to be important measures for mitigating global climate change. Although future sea‐level rise is predicted to change the characteristics of these habitats, its impact on their rate of C_org_ sequestration is highly uncertain. Here we used historical depositional records to show that relative sea‐level (RSL) changes regulated C_org_ accumulation rates in boreal contiguous seagrass–saltmarsh habitats. Age–depth modeling and geological and biogeochemical approaches indicated that C_org_ accumulation rates varied as a function of changes in depositional environments and habitat relocations. In particular, C_org_ accumulation rates were enhanced in subtidal seagrass meadows during times of RSL rise, which were caused by postseismic land subsidence and climate change. Our findings identify historical analogs for the future impact of RSL rise driven by global climate change on rates of C_org_ sequestration in coastal habitats.

## Graphical abstract

Organic carbon (C_org_) storage is one of the important functions of coastal habitats to mitigate climate change; however, the impact of future sea‐level rise on this coastal habitat function is poorly understood. Deposition records in the coastal sediments provide us historical analogs for the future impact of sea‐level rise. Our study in boreal lagoons showed that sea‐level changes altered depositional environments and habitats, affecting C_org_ accumulation rates. C_org_ accumulation rates increased in response to sea‐level rise, indicating that the C_org_ storage function of coastal habitats would provide a negative feedback on climate change.

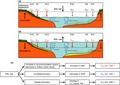

## 1 INTRODUCTION

Fluctuations in relative sea‐level (RSL), which are caused by absolute sea‐level changes and/or absolute movements of the land surface, affect sediment migration and subsequent sediment deposition in coastal waters (Ericson, Vörösmarty, Dingman, Ward, & Meybeck, 2006; Passeri et al., 2015; Syvitski et al., 2009) and shelfs (Molnar, 2004; Schattner, Lazar, Tibor, Ben‐Avraham, & Makovsky, 2010). Sediment deposition directly controls organic carbon (C_org_) burial, which affects carbon budgets at local, regional, and global scales. Enlargement of the space needed to accommodate suspended sediments by RSL rise can potentially increase sediment accretion rates (SARs) if there is a sufficient sediment supply and low hydrological energy (Hori & Saito, 2007; Passeri et al., 2015; Schattner et al., 2010; Woodroffe et al., 2016). However, rapid RSL rise leads to an increased frequency of coastal zone inundation and hence increases the risk of coastal erosion and adversely affects SARs (Ericson et al., 2006; Passeri et al., 2015).

Vegetated coastal ecosystems (seagrass meadows, saltmarshes, and mangroves) play important roles as C_org_ sinks (Nellemann et al., 2009). C_org_ sequestration and absorption of atmospheric carbon dioxide are among the valuable functions of these ecosystems in the context of mitigating the adverse effects of global climate change (Duarte, Losada, Hendriks, Mazarrasa, & Marbà, 2013; Kelleway, Serrano et al., 2017; Macreadie et al., 2017; McLeod et al., 2011; Nellemann et al., 2009; Pendleton et al., 2012). Impacts of RSL rise on these coastal habitats are potentially important feedbacks on their carbon cycles (Craft et al., 2009; Kirwan & Megonigal, 2013; Lovelock et al., 2015; McLeod et al., 2011; Orth et al., 2006). Saltmarshes and mangroves can respond to RSL rise and potentially increase SAR (Kirwan & Mudd, 2012; McKee, Cahoon, & Feller, 2007; Mudd, Howell, & Morris, 2009), but these ecosystems are collapsed if their SARs do not keep pace with RSL rise (Cahoon et al., 2003; Kirwan & Megonigal, 2013). Changes of RSL can also affect carbon budgets by relocating coastal habitats (Kelleway, Saintilan et al., 2017; Kelleway et al., 2016; Pérez et al., 2017).

Although an increasing number of studies have examined the impacts of RSL changes on vegetated coastal habitats (Kirwan & Megonigal, 2013; Lovelock et al., 2015; McLeod et al., 2011), the relationship between RSL change and C_org_ accumulation rates (i.e., the process of C_org_ sequestration) is still poorly understood. Previous studies have demonstrated that RSL rise has potentially affected C_org_ accumulation rates in saltmarshes in recent decades (Carnero‐Bravo et al., 2018; Hill & Anisfeld, 2015; Ruiz‐Fernández et al., 2018). Saltwater intrusion due to RSL rise adversely affects C_org_ accumulation rates in freshwater tidal marshes (Jones, Bernhardt, Krauss, & Noe, 2017). However, the impacts of RSL changes on C_org_ accumulation rates have not been studied in subtidal habitats such as seagrass meadows. There is a need to understand the relationship between RSL changes and C_org_ accumulation rates comprehensively on decadal to millennial timescales because the relevant geophysical and biogeochemical processes that control C_org_ accumulation rates depend on the timescales (Breithaupt et al., 2018).

Historical depositional records can contribute to our understanding of the implications of RSL changes on C_org_ accumulation rates. Analyses of tsunami deposits in sediment cores in eastern Hokkaido, Japan, have revealed recurrent seismic land‐level changes that have affected the depositional environment in coastal lagoons (Kelsey et al., 2006; Nanayama et al., 2003; Sawai et al., 2009). Dense seagrass meadows cover the subtidal zones of these lagoons, and saltmarshes surround their shores (Tokoro et al., 2014; Watanabe & Kuwae, 2015a). The sediment cores of these coastal lagoons may thus preserve the history of changes of RSL, depositional environments, habitats, and C_org_ accumulation rates on millennial timescales.

Here, we examined the sediment profiles of geological and biogeochemical characteristics of two coastal lagoons in eastern Hokkaido. Our goal was to determine how changes of RSL, the depositional environment, and habitat affected C_org_ accumulation rates in vegetated coastal ecosystems. Age–depth modeling at multiple timescales and geological and biogeochemical approaches were used to reconstruct changes of the depositional environments over decadal and millennial timeframes. To study habitat relocations, we used isotopic and elemental signatures to infer historical changes in the sources of sedimentary C_org_.

## 2 MATERIALS AND METHODS

### 2.1 Study sites and sample collection

**Figure 1:**
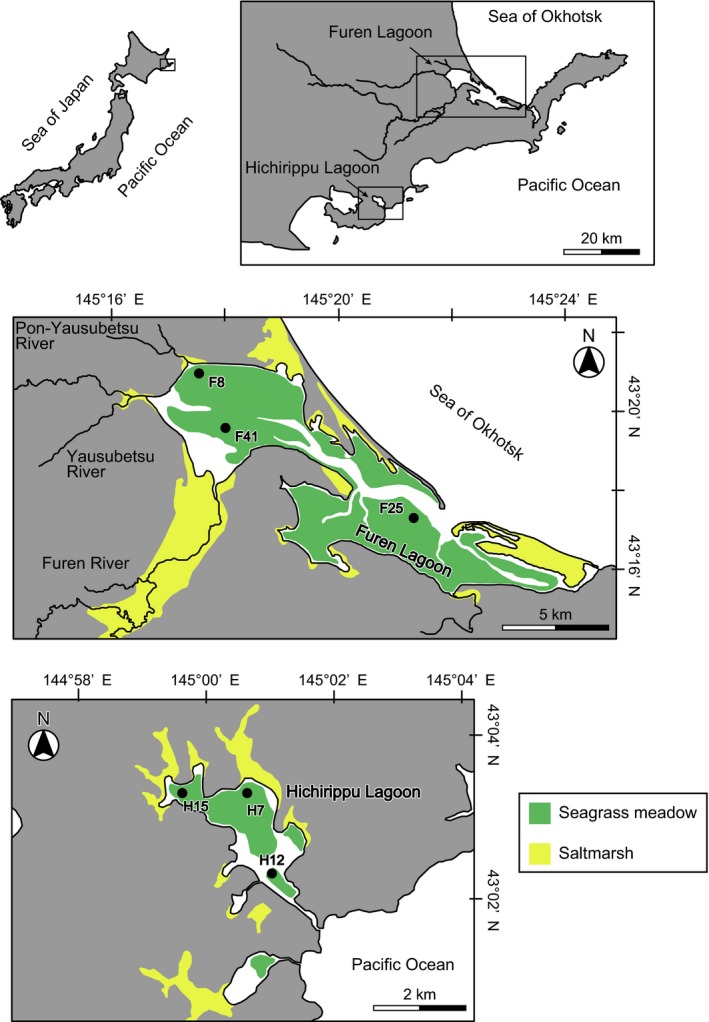
Maps of Furen and Hichirippu lagoons showing the locations of sampling sites. Green and yellow colors represent areas that were occupied by seagrass meadows and saltmarshes, respectively [Colour figure can be viewed at http://www.wileyonlinelibrary.com]

This study was conducted at Furen Lagoon (43°19′46.5″N, 145°15′27.8″E; surface area, 57.4 km^2^) and Hichirippu Lagoon (43°2′44.3″N, 145°0′43.2″E; surface area, 3.56 km^2^) in eastern Hokkaido in Japan (Figure 1). Both of these lagoons are semi‐enclosed systems in the boreal climate region. Silicate and aluminosilicate are the dominant minerals in the sediment of these sites. They have abundant seagrass meadows and are adjacent to surrounding saltmarshes. Because the amount of freshwater input is considerably different between these lagoons, the effect of carbon loads from land could be easily measured.

Furen Lagoon is brackish (salinity, 1–30; Tokoro et al., 2014), and the northern part of the lagoon receives freshwater input from the Furen, Yausubetsu, and Pon‐Yausubetsu rivers (Figure 1). The lagoon is connected to the Sea of Okhotsk. Most of the area is shallow (about 1‐m depth), and *Zostera marina* meadows cover approximately 70% of the total area (Tokoro et al., 2014; Watanabe & Kuwae, 2015a). Three sampling sites (F8, F41, and F25) were chosen inside the *Z. marina* meadow. The topographic features of F8, F41, and F25 are the inner lagoon, the river mouth, and the lagoon mouth, respectively (Figure 1).

The salinity of Hichirippu Lagoon is relatively high (salinity, 27.5–34.1) (Komorita et al., 2014), and the lagoon is connected via a narrow channel to the northwestern Pacific Ocean (Figure 1). The lagoon is shallow (about 0.7‐m depth), and thick seagrass meadows (mainly *Zostera japonica*) cover the bottom. Three sampling sites (H7, H12, and H15) were chosen in the subtidal zone within the *Zostera* meadow. H15 and H7 were located in the inner lagoon, and H12 was located close to the tidal channel.

Six long sediment cores (2‐m length) were collected in October 2012 from seagrass meadows in Furen Lagoon (3 sites) and Hichirippu Lagoon (3 sites) using a hand‐operated knocking corer equipped with a PVC pipe (Adachi, Yamano, Miyajima, & Nakaoka, 2010) or a hand‐operated piston corer. All long cores were sealed at both ends and transported to the laboratory, where they were immediately processed. The compression of cores (defined as the sampled core length divided by the sub‐seafloor penetration of the tube) was usually ≥80%, except for the 0–1 m intervals of F8 (79%), F41 (77%), and F25 (69%), and the 1‐ to 2‐m interval of H15 (78%). We corrected the thickness of each sectioned sample by assuming a linear sediment compression along the core depth during sampling.

Surface sediment cores (in triplicate per site) were also collected using an acrylic piston corer from the same sites. The top 2‐mm sections of the collected surface cores were immediately cut and used to estimate the reservoir effect. The top section was packed into a polyethylene bag and transported to the laboratory at −20°C.

### 2.2 Sample procedures and analyses

After transport to the laboratory, magnetic susceptibility was measured using a Multisensor Core Logger (MSCL‐S; GeoTek Ltd., Daventry, UK) to determine the magnetic mineral concentration of long cores. The core samples were sliced into 1‐cm (the 0‐ to 1‐m interval) or 2‐cm sections (the 1‐ to 2‐m interval). Each sliced sample was kept frozen at −20°C before further analysis.

The sediment grain size of bulk samples was analyzed using a laser‐diffraction/scattering particle size analyzer (LA‐960; HORIBA Ltd., Kyoto, Japan). The sediment samples taken from peaty layers were pretreated with 10% hydrogen peroxide to remove organic matter. Sediments were classified as gravel and coarse sand (>500 µm), medium and fine sand (<500 and >63 µm), and mud (silt and clay, <63 µm).

Samples were prepared by drying sediment (60°C for 24 hr) and measured for net water loss (i.e., water content). The dried sediments were homogenized and stored for analysis of the concentration and stable isotope ratio of organic carbon (C_org_) and nitrogen (TN), dating, and estimation of the dry bulk density (DBD).

Before measurement of the concentration of C_org_ and TN and the stable carbon isotope ratio, the dried sediment was washed with a 1 N HCl solution and dried again at 60°C to remove inorganic carbon. C_org_ and TN concentrations and stable carbon isotope ratios were simultaneously measured with an elemental analyzer‐isotope ratio mass spectrometer (Flash EA 1112/Conflo III/DELTA Plus Advantage; ThermoFisher Scientific, Inc., Bremen, Germany). The stable isotope ratio was expressed in conventional δ notation (δ^13^C) as the deviation from reference materials in parts per thousand (‰). PeeDee Belemnite was used as the reference materials of carbon. The analytical precision of the system, based on the standard deviation of multiple reference replicates (L‐Histidine [δ^13^C‐PDB = −10.18‰; Shoko Co., Ltd., Minato‐ku, Tokyo, Japan]; l‐Alanine [δ^13^C‐VPDB = −19.6‰; Shoko Science Co., Ltd., Yokohama, Kanagawa, Japan]), was normally within ±2% for the concentrations of C_org_ and TN and ±0.2‰ for δ^13^C.

### 2.3 ^210^Pb and ^14^C dating

Sediment accretion rates were determined from ^210^Pb profiles for recent sediments (<100 year BP) and with radiocarbon (^14^C) dating for older sediments. Total ^210^Pb concentrations of dried sediments were analyzed by gamma spectrometry using high‐purity germanium well detectors (GCW3523; Canberra, CT, USA) with a low‐background noise lead chamber (777B, Canberra). The concentrations were determined from the 46.5 keV peak by counting for more than 24 hr. Detector efficiencies for this geometry were calculated using a natural sediment standard (IAEA‐375). Supported ^210^Pb (^210^Pb_sup_) at each core was estimated as the average ^210^Pb concentration of the deeper sections where ^210^Pb reached constant concentrations. Excess ^210^Pb (^210^Pb_ex_) concentrations were calculated by subtracting the ^210^Pb_sup_ from the total ^210^Pb (Supporting Information Figure S1). The date of each section was determined from a constant‐rate‐of‐supply (CRS) model based on the ^210^Pb_ex_ inventory (Appleby & Oldfield, 1978).

For ^14^C dating, more than 12 sections from each long sediment core and the top 2‐mm sections of the surface cores were analyzed. The bulk dried sediments were treated to remove inorganic carbon (i.e., acid washed) and plant debris, and the remaining C_org_ was combusted in an elemental analyzer (either a Euro EA3000; EuroVector, Milan, Italy; or a Flash 2000; Thermo Fisher Scientific, Inc., Waltham, MA, USA). The evolved CO_2_ was collected cryogenically, purified in a vacuum line, and reduced to graphite using hydrogen and an iron catalyst at 650°C for 10 hr. Then, the ^13^C and ^14^C abundances were determined using accelerator mass spectrometry (AMS). The conventional radiocarbon age was converted into a calendar date in years before present (Cal year BP; present taken as AD 2012) using the OxCal 4.3 (IntCal13 dataset; Reimer et al., 2013). We estimated the marine reservoir effect (Δ*R*) as the average date of the top 2‐mm sections of each site because the composition of C_org_ sources differed among the sites.

Age–depth models for all cores were constructed using the Bayesian age–depth modeling software “Bacon” (Blaauw & Christen, 2011), with ^210^Pb (CRS model) and ^14^C dates. The mean age of each sediment slice was estimated from the age–depth models.

### 2.4 C_org_ stock and accumulation rate estimation

C_org_ stocks were determined from the C_org_ concentrations and DBDs. The DBD of a sediment sample was calculated using the water content (*w*, g/g) of each sample and the average grain density (*d*, g/cm^3^) determined for some representative samples in each core. The value of *d* was determined using pycnometers with pure water. The average *d* was 2.48–2.66 g/cm^3^. The DBDs of the sediment samples were calculated as follows:

$$\text{DBD}\;\left({\text{g/cm}}^{3}\right)\;=\;\frac{1\;-\;1.023w}{w+\;\left(1\;-\;1.023w\right)/d},$$

where the pore water density was assumed to be 1.023 g/cm^3^ (salinity 35 at 25°C). C_org_ density (g C_org_/cm^3^) was calculated using C_org_ concentration and DBD for each sediment slice. The C_org_ stocks (g C_org_/m^2^) were estimated by calculating the cumulative mass of C_org_ accumulated over multiple timescales.

Averaged C_org_ accumulation rates for the decadal (<100 years) timescales were calculated from linear regression of the cumulative C_org_ stocks vs. the ^210^Pb dates. Averaged C_org_ accumulation rates for millennial (>1,000 years) timescales were calculated from linear regression of the cumulative C_org_ stocks vs. the ^14^C dates (Cal year BP). C_org_ accumulation rates for each sediment slice were also estimated as the slope of the linear regression between the cumulative C_org_ stocks and the dates of the age–depth model using the three peripheral slices.

### 2.5 C_org_ source mixing model

The Bayesian isotopic modeling package, SIAR (Parnell, Inger, Bearhop, & Jackson, 2010), was used to partition the contributions of potential sources to the sedimentary C_org_ based on their N/C and δ^13^C signatures. We chose N/C rather than C/N ratios in the model because the former was statistically more robust (Perdue & Koprivnjak, 2007; Watanabe & Kuwae, 2015a). For each potential source, we report the median and the 95% credible interval of the estimate of the proportional contribution of each source.

For the Furen Lagoon model, we defined five potential C_org_ sources, that is, seagrass, phytoplankton, microphytobenthos (MPB), saltmarsh plants, and riverine particulate organic matter (riverine OM) as endmembers. To suppress the divergence of the estimation results, we pooled phytoplankton and MPB as microalgae, and we also pooled saltmarsh plants and riverine OM as terrestrial organic matter (TerrOM) (Supporting Information Figure S2). For the Hichirippu Lagoon model, seagrass, microalgae, and TerrOM (saltmarsh plants + riparian soil) were considered as potential C_org_ sources (Supporting Information Figure S2).

Seagrass was collected seasonally from several sites located along the salinity gradient in Furen Lagoon (in 2014) and Hichirippu Lagoon (from 2004 to 2008). The individual plant values were determined as the average values of aboveground and belowground parts. Particulate organic carbon samples collected in Furen Lagoon (in 2014) and Hichirippu Lagoon (from 2005 to 2007), which were characterized by low POC/chlorophyll‐*a* ratios (<100) and low C/N ratios (<8.0), were considered to be representative of phytoplankton (Watanabe & Kuwae, 2015a). Because previous studies have reported that the N/C ratios of terrestrial plants with extremely low N/C ratios changed during decomposition (Krüger, Leifeld, Glatzel, Szidat, & Alewell, 2015), saltmarsh detritus was sampled directly from the peat layers in cores H7 and H15, and then, the average value was determined. Riparian soil (top 5 mm) was collected using a 2.9‐cm‐diameter corer in the Hichirippu Lagoon watershed. N/C and δ^13^C for sources were analyzed following the method described above.

Riverine OM was determined as the average value of samples taken at the three river mouths (salinity = 0) in Furen Lagoon in 2014 (Watanabe & Kuwae, 2015b). The values of N/C and δ^13^C for MPB were taken from values in the literature for Furen Lagoon (Kuwae et al., 2012) and Hichirippu Lagoon (Kajihara et al., 2010). Microalgae was defined as a mixture of phytoplankton and MPB, and TerrOM was likewise defined as a mixture of saltmarsh plants and riverine OM/riparian soil. The signature values were computed using a linear interpolation and a mixing ratio that ranged from 0% to 100%.

Because the N/C ratio generally declines while OM is decomposing (Krüger et al., 2015), the contribution from the source with a high‐N/C (i.e., microalgae) to sedimentary C_org_ was probably underestimated. In contrast, there is little change of δ^13^C during decomposition (Fry, 2006).

### 2.6 Statistical analyses

Statistical analyses were performed using R statistical packages (R Core Team, 2017). We used a generalized linear model with a gamma distribution and identity link to examine the effects of mineral mass accumulation rate (MAR), mud content, and sediment type (peat/subtidal) on C_org_ accumulation rate. The best model was selected according to the Akaike Information Criterion. Mineral density in each sediment sample was calculated with the following equations:

$$\text{Mineral density}\;\left({\text{g/cm}}^{3}\right)\;=\;\text{DBD}\;\times \;\left(1\;-\;\text{organic matter content}\right)$$

Organic matter content was defined as loss on ignition (LOI), the fractional weight loss of dry sediment samples after combustion at 500–550°C. In this study, LOI was estimated from C_org_ concentration using the linear regressions between these parameters (Fourqurean et al., 2012) as follows:

$$\text{LOI}\;\left(\%\right)\;=\;\frac{{\text{C}}_{\text{org}}\left(\%\right)\;+\;0.21}{0.4}\left(\text{LOI}\;<\;20\%\right)$$

$$\text{LOI}\;\left(\%\right)\;=\;\frac{{\text{C}}_{\text{org}}\left(\%\right)\;+\;0.33}{0.43}\left(\text{LOI}\;\geq \;20\%\right)$$

Mass accumulation rate was estimated as the slope of the linear regression between the cumulative mineral mass and the dates of the age–depth model using the three peripheral slices.

Change point analysis (Killick & Eckley, 2014) was applied to the vertical profiles of the C_org_ accumulation rates to identify the time periods of rapid C_org_ accumulation. Hierarchical clustering analysis was used to classify the depositional environment of the time periods of rapid C_org_ accumulation. We carried out the clustering analysis based on the Euclidean distance and Ward criterion using the C_org_ concentrations, grain size composition, and C_org_ source composition of the sediment samples identified by the change point analysis. This dataset was also used for principal component analysis.

## 3 RESULTS

### 3.1 C_org_ stocks and accumulation rates

The SARs at the decadal timescale were estimated to be 0.6–4.9 and 0.2–1.9 mm/year in Furen Lagoon and Hichirippu Lagoon, respectively (Table 1). Based on the ^14^C dates (Cal year BP), the averaged SARs at the millennial timescale were estimated to be 0.2–1.2 and 0.4–0.6 mm/year in Furen Lagoon and Hichirippu Lagoon, respectively (Table 1). The decadal scale SARs were significantly higher than those at the millennial timescale except for cores F41 and H7 (*t* test, *p* < 0.01).

**Table 1:** Average sediment accretion rates (SARs) and C_org_ accumulation rates in each site (mean ± 95% confidence interval)

Site	Contemporary habitat and geomorphological setting	Decadal scale (^210^Pb)			Millennial scale (^14^C)			
		Thickness (cm)	SAR (mm/year)	C_org_ acc. rate (g C_org_ m^−2^ year^−1^)	Thickness (cm)		SAR (mm/year )	C_org_ acc. rate (g C_org_ m^−2^ year^−1^)
Furen Lagoon								
F8	*Zostera marina* Inner lagoon	8	1.0 ± 0.2	11.7 ± 3.5	200	0.2 ± 0.0		2.0 ± 0.4
F41	*Z. marina* River mouth	6	0.6 ± 0.2	7.5 ± 3.1	200	0.6 ± 0.1		5.1 ± 1.0
F25	*Z. marina* Lagoon mouth	30	4.9 ± 1.0	41.6 ± 7.7	200	1.2 ± 0.5		11.8 ± 4.5
Hichirippu Lagoon								
H15	*Zostera japonica* Inner lagoon	20	1.9 ± 0.4	31.0 ± 7.0	200		0.6 ± 0.1	14.8 ± 3.8
H7	*Z. japonica* Inner lagoon	2	0.2 ± 0.1	5.1 ± 5.6	200		0.5 ± 0.1	9.2 ± 3.2
H12	*Z. japonica* Edge of tidal channel	6	1.5 ± 0.1	11.7 ± 4.0	200		0.4 ± 0.1	2.7 ± 0.6

Note

Decadal (derived from ^210^Pb dating) and millennial (derived from ^14^C dating) rates are estimated.

The decadal scale C_org_ accumulation rate in core F25 (41.6 g C_org_ m^−2^ year^−1^) was higher than those at the inner sites (F8 and F41, 11.7 and 7.5 g C_org_ m^−2^ year^−1^, respectively) in Furen Lagoon (Table 1). The highest millennial‐scale C_org_ accumulation rate was also found in core F25 (11.8 g C_org_ m^−2^ year^−1^). In Hichirippu Lagoon, both the decadal‐ and millennial‐scale C_org_ accumulation rates in core H15 (31.0 and 14.8 g C_org_ m^−2^ year^−1^) were higher than those at the other sites in Hichirippu Lagoon, which was comparable to those in core F25 (Table 1). The decadal scale C_org_ accumulation rates were significantly higher than those at the millennial scale except for cores F41 and H7 (*t* test, *p* < 0.01).

### 3.2 Geological and biogeochemical features of sediment cores

**Figure 2:**
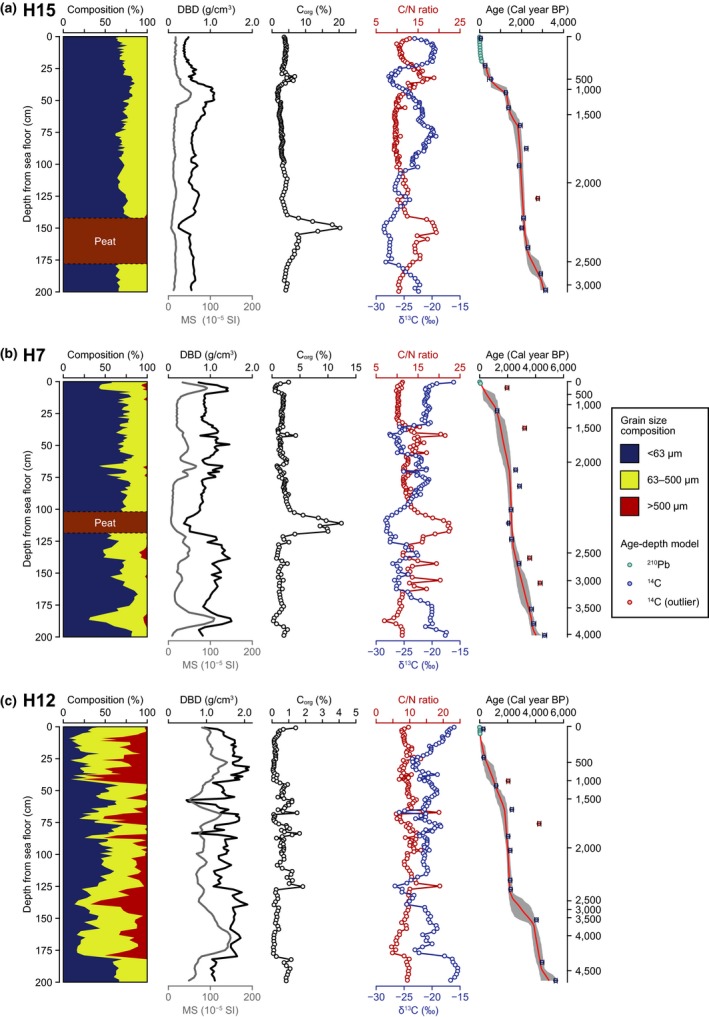
Vertical profiles of grain size composition, dry bulk density (DBD), magnetic susceptibility (MS), organic carbon (C_org_) concentration, C/N ratio, δ^13^C, and age–depth model at sampling sites in Hichirippu Lagoon. In the age–depth model, the red lines and gray areas represent the weighted mean estimates and 95% credible intervals, respectively [Colour figure can be viewed at http://www.wileyonlinelibrary.com]

In the cores of Hichirippu Lagoon, SARs temporally changed, which was linked with the changes in other biogeochemical signatures (Figure 2). In core H15, the peaty sediment layer was distributed at 140‐ to 175‐cm depth (around 2,400 Cal year BP), which represented high C_org_ concentration (~20.3%), high C/N ratio (~20), and ^13^C‐depleted signature (approximately −28.6‰; Figure 2a). The section at 75–140 cm depth showed the rapid SAR with the increase in δ^13^C. From 25 to 75 cm depth, δ^13^C decreased with the slow SAR. The contemporary deposit (approximately 25‐cm depth) was accumulated rapidly with the increase in δ^13^C. In core H7, several sandy layers were deposited inside the dominant muddy sediment (Figure 2b). The peaty sediment at 100‐ to 120‐cm depth was accumulated on the sandy layer, which was high C_org_, ^13^C‐depleted. The section above the peaty layer (75‐ to 100‐cm depth) showed the rapid SAR and the increase in δ^13^C. In the top 75 cm section, C/N ratio and δ^13^C fluctuated and the SAR was relatively slow. The grain size distribution was heterogeneous, and the coarse sand layer was distributed in the muddy sand sediment in core H12 (Figure 2c). The δ^13^C signature was the highest among the sediments of Hichirippu Lagoon. C_org_ concentration was lower than 1% through the profile. The SAR accelerated around 1,900–2,200 Cal year BP at 75‐ to 130‐cm depth.

**Figure 3:**
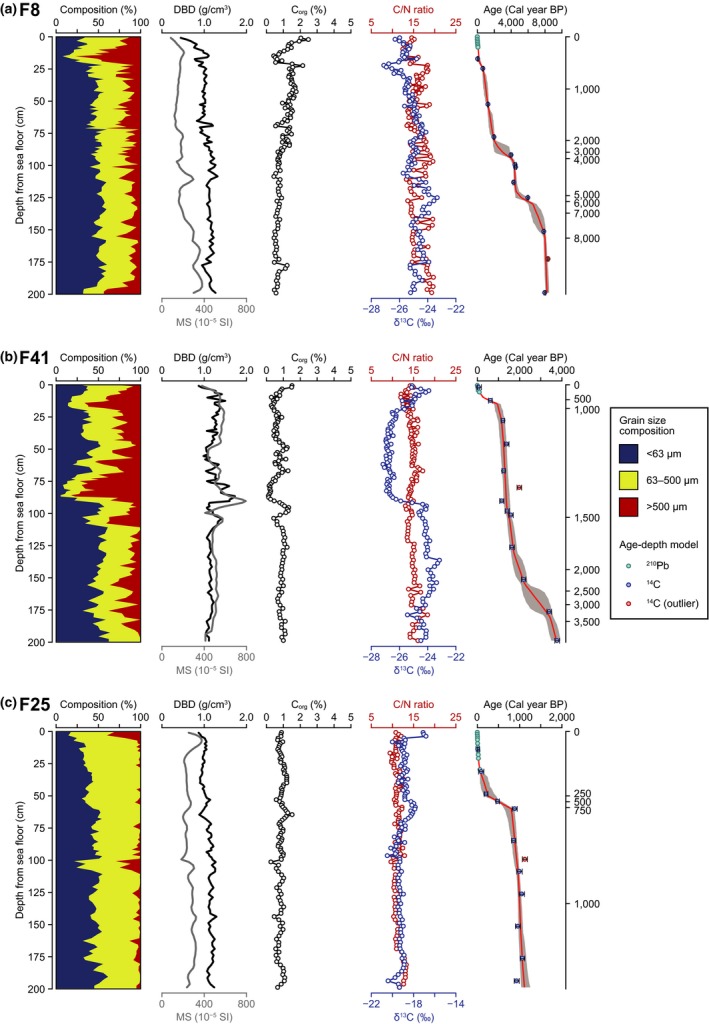
Vertical profiles of grain size composition, dry bulk density (DBD), magnetic susceptibility (MS), organic carbon (C_org_) concentration, C/N ratio, δ^13^C, and age–depth model at sampling sites in Furen Lagoon. In the age–depth model, red lines and gray areas represent the weighted mean estimates and 95% credible intervals, respectively [Colour figure can be viewed at http://www.wileyonlinelibrary.com]

The sediment of Furen Lagoon was mainly composed of muddy sand and sand (Figure 3). The magnetic susceptibility was considerably higher in Furen Lagoon than Hichirippu Lagoon. Fine sand and mud dominated the sediment but coarse sand layer was found at 10–20 cm depth in core F8 (Figure 3a). C_org_ concentration was more than 2% at the sediment surface, and decreased <1% to the deeper sediments. C/N ratio was ranging from 12 to 20. δ^13^C was relatively stable but slightly fluctuated in a range of −23.3‰ to −27.1‰. In core F41, C_org_ concentrations were lower than 1.5% almost through the profile, and especially low at coarse sand layer (Figure 3b). C/N ratio and δ^13^C were in ranges of 12–18 and −23.1‰ to −27.2‰. In core F25, the sediment was dominated by fine and medium sand (Figure 3c). C_org_ concentration was stable and almost lower than 1%. C/N ratio (9–14) was lower than the other sites in Furen Lagoon. δ^13^C ranged from −20.5‰ to −16.8‰, which was the most ^13^C‐enriched sediment in Furen Lagoon sites.

### 3.3 Composition of C_org_ sources in sediments

**Figure 4:**
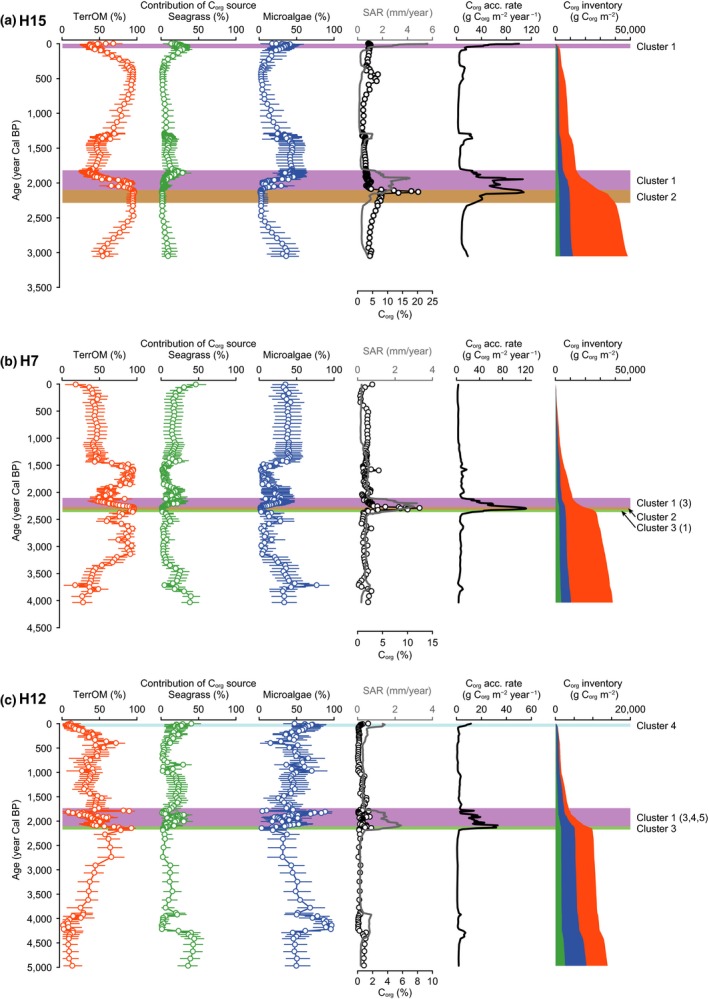
Historical records from Hichirippu Lagoon cores. Temporal changes in the contribution of each organic carbon (C_org_) source, C_org_ concentration, sediment accretion rate (SAR), C_org_ accumulation rate, and C_org_ inventories in H15 (a), H7 (b), and H12 (c). Color shadings mark the time periods of rapid C_org_ accumulation. These periods were classified with a hierarchical clustering analysis based on C_org_ concentration, grain size composition, and C_org_ source composition of the sediment samples. In addition to the major clusters, minor clusters were shown in parentheses. Error bars show the 95% credible intervals of the estimates [Colour figure can be viewed at http://www.wileyonlinelibrary.com]

**Figure 5:**
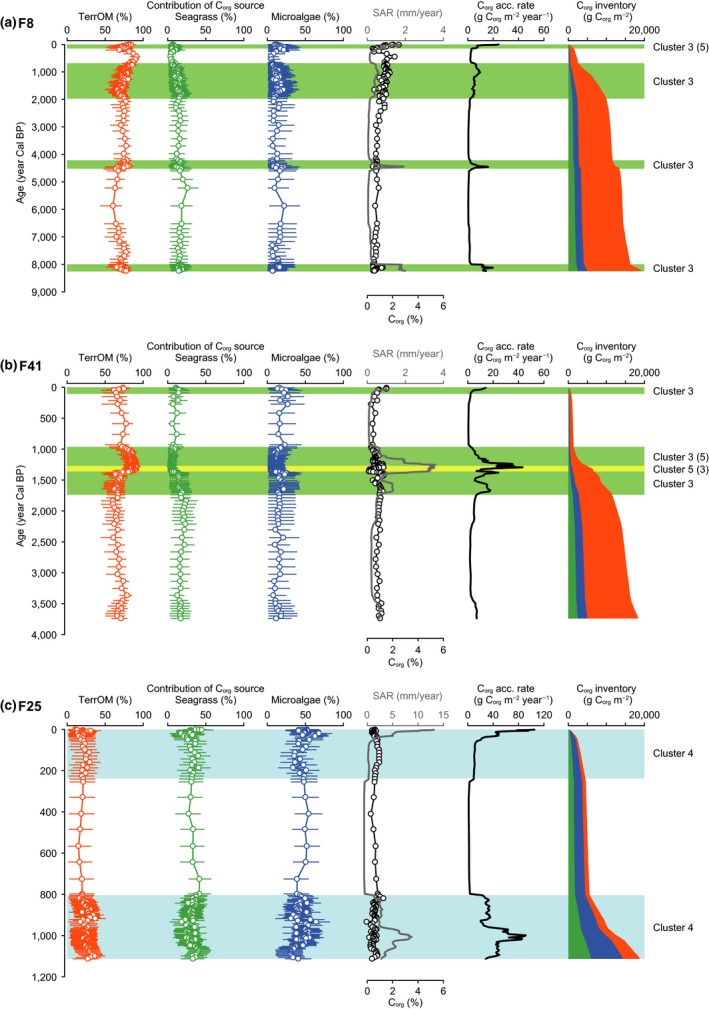
Historical records from Furen Lagoon cores. Temporal changes in the contribution of each organic carbon (C_org_) source, C_org_ concentration, sediment accretion rate (SAR), C_org_ accumulation rate, and C_org_ inventories in F8 (a), F41 (b), and F25 (c). Color shadings mark the time periods of rapid C_org_ accumulation. These periods were classified with a hierarchical clustering analysis based on C_org_ concentration, grain size composition, and C_org_ source composition of the sediment samples. In addition to the major clusters, minor clusters were shown in parentheses. Error bars show the 95% credible intervals of the estimates [Colour figure can be viewed at http://www.wileyonlinelibrary.com]

The C_org_ source mixing model showed the temporal and spatial variation in the C_org_ sources in the sediment of the coastal vegetated habitats (Figures 4 and 5; Supporting Information Figure S2). The dominant C_org_ sources temporally changed in the sediment cores collected in Hichirippu Lagoon (Figure 4). TerrOM occupied more than 90%, and the contributions of seagrass and microalgae were near 0 at the peaty layers in cores H15 and H7. The contribution of microalgae and seagrass increased at the upper section of the peaty layers. In core H12, the peaty layer was not distributed but the contribution of TerrOM was high around 2,400 Cal year BP. In Furen Lagoon, TerrOM was the most dominant source in cores F8 and F41 (Figure 5). The average contributions of TerrOM were 79% (78%–81%, 95% credible interval) and 79% (76%–81%) in cores F8 and F41, respectively. In these cores, seagrass and microalgae were less important sources of C_org_ than TerrOM. The average contribution of TerrOM was significantly lower in the F25 sediment (20%–24%, 95% CI) than at the inner sites (Figure 4). Seagrass (29%–32%) and microalgae (46%–49%) were the dominant components in the F25 sediment.

### 3.4 Historical changes in C_org_ accumulation rates

The age–depth model showed that C_org_ accumulation rates had spatial and temporal variations (Figures 4 and 5). The sediment samples identified as the time periods of rapid C_org_ accumulation were classified into five clusters (Supporting Information Figure S3). Cluster 1 was classified as the muddy sediment composed of TerrOM, seagrass, and microalgae. Cluster 2 was classified as the peaty sediment with a high C_org_ content. Cluster 3 was the sandy sediment which was dominated by TerrOM. Cluster 4 was the sandy sediment with a high contribution of seagrass and microalgae. Cluster 5 was the sediment containing coarse sand and grain.

**Figure 6:**
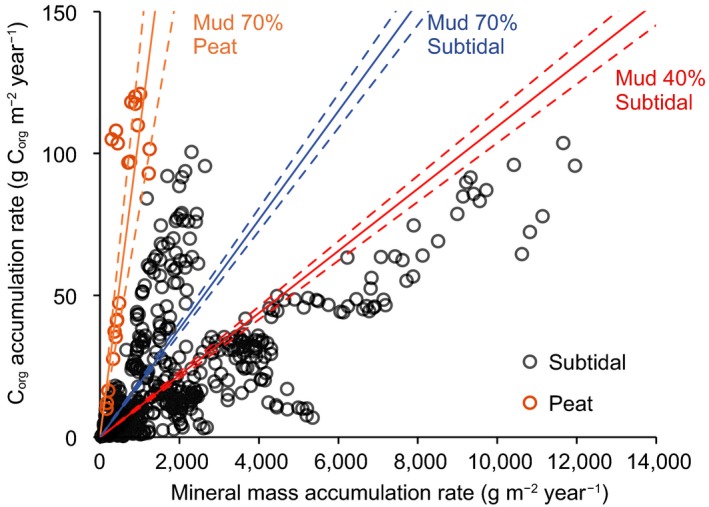
Relationships between organic carbon (C_org_) accumulation rate, mineral mass accumulation rate, mud (<63 µm) content, and sediment type (peat/subtidal) analyzed with a generalized linear model. The solid and dashed lines indicate the linear models and their 95% confidence intervals, respectively [Colour figure can be viewed at http://www.wileyonlinelibrary.com]

In cores H15 and H7, C_org_ accumulation rates were high in the peaty layers (Cluster 2) which were deposited around 2,400 Cal year BP. Average C_org_ accumulation rates were 59.0 and 117 g C_org_ m^−2^ year^−1^ in H15 and H7, respectively, during this period (Figure 4). C_org_ accumulation rates were high from 2,400 Cal year BP to around 1,800 Cal year BP (Cluster 1), with the averages of 56.2, 46.1, and 13.1 g C_org_ m^−2^ year^−1^ in H15, H7, and H12, respectively (Figure 4). In H15 and H12, the C_org_ accumulation rates in the last century increased markedly. At the inner lagoon sites of Furen Lagoon, the average C_org_ accumulation rates around 1800 Cal year BP to 800 Cal year BP (F8, 5.8 g C_org_ m^−2^ year^−1^; F41, 15.0 g C_org_ m^−2^ year^−1^) were higher than the millennial‐scale accumulation rates (Table 1; Figure 5). C_org_ accumulation rates from 1,100 to 800 Cal year BP (43.9 g C_org_ m^−2^ year^−1^) were higher at F25 than at the inner sites (Figure 5). In contrast, C_org_ accumulation was inhibited from 800 to 200 Cal year BP in Furen Lagoon. A generalized linear model supported that both MARs and mud contents significantly control C_org_ accumulation rates (Figure 6; Table 2).

**Table 2:** Generalized linear model result for the effect of mineral mass accumulation rate (MAR), mud content, and sediment type (peat/subtidal) on organic carbon (C_org_) accumulation rate

	Estimate	*SE*	*t*	*P* (>*t*)
C_org_ acc. rate ~ MAR × mud × type, family = Gamma, AIC = 4,431.5				
Intercept	−0.09279	0.07965	−1.165	0.244
MAR × mud × peat	0.001548	0.0002034	7.61	<0.0001
MAR × mud × subtidal	0.000274	0.000007499	36.537	<0.0001

Note

The best model according to its Akaike information criterion (AIC) value is shown.

## 4 DISCUSSION

### 4.1 Historical depositional environment in the contiguous seagrass–saltmarsh habitats

This study was the first to examine the C_org_ storage function of vegetated coastal ecosystems in the boreal region of the northwestern Pacific (Supporting Information Appendix S1). The elucidation of historical and spatial variations of C_org_ sources in the sediments of the vegetated coastal habitats using isotopic and elemental composition data with a C_org_ source mixing model showed that these sediment cores recorded historical changes in depositional environments and habitats. The dominant C_org_ sources fluctuated over time in the Hichirippu Lagoon sediment cores (Figure 4). Around 2,400 Cal year BP, microalgae‐dominated and low‐C_org_ deposits became high C_org_ deposits dominated by terrestrial‐derived organic matter (TerrOM). Regarding the formation of these peaty layers (Figure 2), Sawai et al. (2009) found peaty deposits distributed on a tsunami deposit and have suggested that seismic land uplift abruptly transformed a subtidal environment into a swamp around 2,400 Cal year BP in a lagoon located beside Hichirippu Lagoon. The profile of core H7 also revealed a transition from a sandy layer to an overlying peaty sediment at a depth of 120–140 cm at an estimated time of 2,400 Cal year BP. Although a sandy deposit was not found around 2,400 Cal year BP in core H15, peaty sediment was identified at almost the same time. Peaty sediment was not found in core H12, but the contribution of terrestrial‐derived C_org_ increased above a coarse sand layer (depths of 126–140 cm, Figure 2c) around 2,500 Cal year BP. These changes in depositional environments occurred simultaneously, driven by a seismic land uplift (Sawai et al., 2009).

The contributions of microalgae and seagrass increased in the upper section of the peaty layers (Figure 4). The reestablishment of a subtidal environment soon after the peaty sediment was deposited in Hichirippu Lagoon demonstrated that the saltmarsh was submerged by the RSL rise. This RSL rise occurred locally and was driven by postseismic land subsidence (Sawai, Horton, & Nagumo, 2004; Sawai et al., 2009). Increases of δ^13^C values and the contributions of microalgae and seagrass‐derived C_org_ from 2,400 to 1,800 Cal year BP indicated that the RSL rise progressed during this period (Figures 2 and 4).

We also found some changes in depositional environments that could be related to the RSL change in three Furen Lagoon cores, although the apparent emergence event found in Hichirippu Lagoon was not identified (Figures 3 and 5). Terrestrial‐derived C_org_ was the most abundant form of C_org_ in the cores collected in the inner part of the lagoon (i.e., F8 and F41). In these cores, seagrass and microalgae were less important sources of C_org_ than terrestrial‐derived C_org_. SARs were high from around 1,800 to 800 Cal year BP and during the last century in both F8 and F41 (Figure 5). A previous study identified four seismic uplift events during the past 2,000 years: 300, 400–600, 1,400, and 1,900 Cal year BP (Kelsey et al., 2006). The periods of rapid accretion could be linked with the inter‐seismic subsidence and RSL rise. In the F41 core profile, the clear decline of δ^13^C values from 1,400 to 1,000 Cal year BP (depths of 18–90 cm, Figure 3b) indicated that terrestrial‐derived C_org_ was the dominant source of C_org_ during this period. The lower part of this depositional unit was occupied by coarse sand with high magnetic susceptibility, and the sediment grain size became finer upward. These results indicate that fluvial deposits accumulated rapidly around 1,400 Cal year BP. In core F25, sediment accumulated rapidly around 800–1,100 Cal year BP and during the last 100 years (Figure 5c), also corresponding with the periods of inter‐seismic RSL rise (Kelsey et al., 2006).

### 4.2 Impact of RSL changes on C_org_ accumulation rates

**Figure 7:**
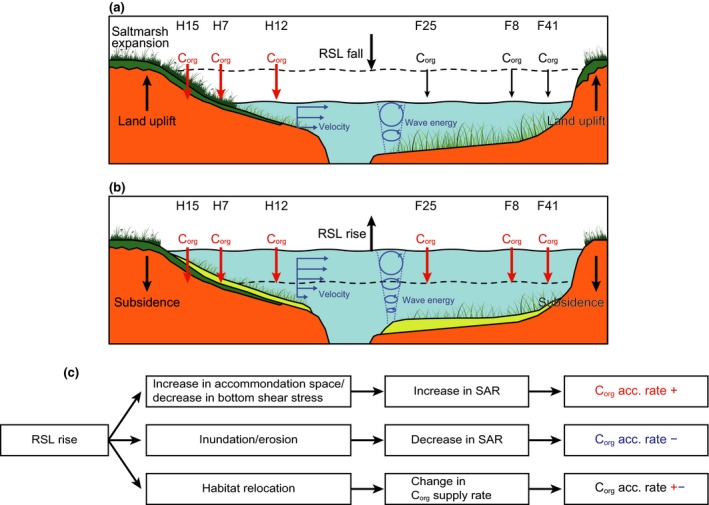
Conceptual diagram of the impact of relative sea‐level (RSL) changes on C_org_ accumulation rates in contiguous seagrass–saltmarsh habitats. In inner lagoonal habitats, expansion of saltmarsh habitats to the deeper zone as RSL falls induces the rapid deposition of saltmarsh‐derived C_org_ in the supratidal and intertidal zones (a). RSL rise induces saltmarsh inundation, which relocates saltmarsh habitats to seagrass meadows (b). While RSL rises, the expansion of space to accommodate suspended sediment and the decrease in bottom shear stress accelerate multi‐source C_org_ accumulation rates in the subtidal zone (b). Flow diagram of impacts of RSL rise on C_org_ accumulation rates (c) [Colour figure can be viewed at http://www.wileyonlinelibrary.com]

On both decadal and millennial timeframes, C_org_ accumulation rates were controlled largely by sediment accretion, which has fluctuated historically in Hichirippu and Furen lagoons (Figures 4, 5, 6; Table 2). Vertical analysis of the geological and biogeochemical features showed that changes of C_org_ accumulation rates were related to historical changes of the depositional environment in eastern Hokkaido. The increase of decadal (the last century) C_org_ accumulation rates in both lagoons could be explained by RSL rise (Figure 7). Geodetic measurements have shown that submergence (including land subsidence and recent sea‐level rise due to global climate change) averaged 5–10 mm/year during the past century along the Pacific coast of eastern Hokkaido (Atwater et al., 2004). The SAR would be increased by the greater depths of the water columns in both lagoons, but the SARs varied among sites as functions of erosion rates and sediment loading. The C_org_ accumulation rate was significantly higher at the decadal scale than at the millennial scale, except for cores H7 and F41. A caveat to this conclusion is that the dating methods and the cumulative amount of degradation differed for the decadal‐ and millennial‐scale analyses, and sediment compression may have caused the C_org_ accumulation rate in the upper sediment to be overestimated relative to the older sediment (Breithaupt et al., 2018). The depositional environment during the last century, however, would have favored rapid C_org_ accumulation.

At the millennial timescale, C_org_ accumulation rates were high during expansion of the saltmarsh due to land uplift around 2,400 Cal year BP in Hichirippu Lagoon. The high C_org_ accumulation rates were supported by the rapid supply of saltmarsh debris, which contained high C_org_ (Figure 6; Table 2). The C_org_ accumulation rate was also enhanced by the RSL rise that occurred after submergence of the saltmarsh in Hichirippu Lagoon. The habitat shift from saltmarsh to subtidal zone (i.e., seagrass meadow) showed that the saltmarsh was not able to maintain its elevation above sea level, despite the enhanced sediment accretion in response to RSL rise. Rapid sediment accretion was responsible for the rapid C_org_ accumulation under subtidal conditions (Figure 4). The low magnetic susceptibility of these deposits suggests that the SAR was enhanced by the greater water depth and associated attenuation of wave energy and bottom velocities but not by an abrupt increase in riverine sediment load (Figure 7) (Collins et al., 2017; Morris, Sundareshwar, Nietch, Kjerfve, & Cahoon, 2002; Woodroffe et al., 2016).

In Furen Lagoon, rapid sediment accretion corresponding to RSL rise enhanced C_org_ accumulation. C_org_ accumulation rates from 1,100 to 800 Cal year BP were higher at F25 than at the inner sites. Because F25 was located close to the barrier spits of the lagoon mouth, the abundant sediment supply from outside would have contributed to the high rate of C_org_ accumulation (Theuerkauf & Rodriguez, 2017). In contrast, C_org_ accumulation was inhibited from 800 to 200 Cal year BP in Furen Lagoon. The fall of RSL that occurred with land uplift during this period (Kelsey et al., 2006) would have enhanced sediment erosion and reduced C_org_ accumulation rates.

Our age–depth model and C_org_ source estimates showed that RSL changes affected depositional environments, relocated habitats, and consequently regulated C_org_ accumulation rates in vegetated coastal habitats on decadal to millennial timescales. In particular, the enhancement of C_org_ accumulation rates by raising of RSL in subtidal zones (Figure 7) indicates that this process has a negative feedback on climate change. Previous studies have reported that supratidal and intertidal vegetated habitats and lagoons can potentially maintain their elevations in response to enhanced sea‐level rise (McKee et al., 2007; Mudd et al., 2009; Theuerkauf & Rodriguez, 2017). However, if the sediment load or root growth is insufficient to maintain elevation above sea level, these habitats can be eroded and submerged (Cahoon et al., 2003; Kirwan & Mudd, 2012; McLeod et al., 2011; Mudd et al., 2009). Our sedimentological records demonstrate that the intertidal saltmarsh was flooded due to rapid sea‐level rise (>2.7 mm/year, Figure 4) and shifted to subtidal seagrass meadows in Hichirippu Lagoon. The subsequent rapid burial of the saltmarsh‐derived C_org_‐rich sediment created an anoxic layer that constrained C_org_ decay and contributed to long‐term preservation of C_org_ (McLeod et al., 2011). However, the habitat transformation from saltmarsh to seagrass meadow negatively affected the C_org_ accumulation rate. Future sea‐level rise is predicted to increase the risk of saltmarsh inundation (Craft et al., 2009; Kirwan & Megonigal, 2013), which would relocate saltmarsh habitats to subtidal seagrass meadows and reduce the rate of C_org_ sequestration.

Previous studies have demonstrated that sea‐level rise stimulates the primary production of saltmarsh plants (Morris, Sundberg, & Hopkinson, 2013) and thereby increases C_org_ accumulation rates (Hill & Anisfeld, 2015). However, the present study shows that RSL rise increases C_org_ accumulation rates by enhancing sediment accretion in subtidal seagrass meadows. Similar response is found in saltmarshes at the decadal timescale (Ruiz‐Fernández et al., 2018). By expanding the area where sedimentation can occur (Collins et al., 2017; Morris et al., 2002; Woodroffe et al., 2016) and decreasing bottom shear stress, RSL rise enhances sediment accretion in subtidal zones (Figure 7). The high SARs during RSL rise in seagrass meadows close to the mouth of Furen Lagoon contributed to the high C_org_ accumulation rates. Sediment accretion rates may therefore respond more strongly to sea‐level rise in geomorphological settings where sediment loads are abundant. The variable impact of RSL rise on C_org_ accumulation is a function of geomorphological settings, habitats, and hydrodynamic conditions (Figure 7). Furthermore, land‐level subsidence locally enhances the influence of sea‐level rise (Shields et al., 2017). Future studies concerned with the variability of these factors are needed to enhance understanding of the impact of RSL changes on the functionality of vegetated coastal habitats and thereby inform coastal conservation and restoration strategies to mitigate climate change impacts.
