# Supplementary material for: Relative sea‐level change regulates organic carbon accumulation in coastal habitats
Source: Glob Chang Biol. 2019 Jan 24;25(3):1063–77. doi: 10.1111/gcb.14558 (PMC6850580; doi:10.1111/gcb.14558)
Supplement: Supplementary file 1 [file GCB-25-1063-s001.pdf]

Supporting information for

Relative sea-level change regulates organic carbon accumulation in coastal habitats

Kenta Watanabe[1,*], Koji Seike[2], Rumiko Kajihara[3], Shigeru Montani[4] and Tomohiro Kuwae[1]

[1]Coastal and Estuarine Environment Research Group, Port and Airport Research Institute, 3-1-1 Nagase, Yokosuka 239-0826, Japan

[2]Geological Survey of Japan, National Institute of Advanced Industrial Science and Technology (AIST), Central 7, 1-1-1 Higashi, Tsukuba 305-8567, Japan

[3]Civil Engineering Research Institute for Cold Region, 1-3-1-34 Hiragishi, Toyohira-ku, Sapporo 062-8602, Japan

[4]Graduate School of Environmental Science, Hokkaido University, N10W5 Kita-ku, Sapporo 060–0810, Japan

*Corresponding author. e-mail: watanabe-ke@p.mpat. go.jp

Appendix S1. Organic carbon accumulation rates in eastern Hokkaido

This study is the first to assess the organic carbon (C_{org}) storage function of vegetated coastal habitats in the boreal region of the northwestern Pacific Ocean. We compared both the decadal- and millennial-scale average C_{org} accumulation rates in Furen and Hichirippu lagoons with the rates at other sites (Figure 1).

The decadal-scale C_{org} accumulation rates (estimated by ^{210}Pb) in this study ranged from 5.1 to 41.6 g C_{org} m^{-2} yr^{-1} (Table 1), which is comparable with the rates in other *Zostera* meadows (0.8–41 g C_{org} m^{-2} yr^{-1}) (Greiner et al., 2013; Jankowska et al., 2016; Postlethwaite et al., 2018), *Posidonia* meadows (9–52 g C_{org} m^{-2} yr^{-1}) (Mazarrasa et al., 2017; Serrano et al., 2016), and saltmarshes (10–80 g C_{org} m^{-2} yr^{-1}) (Kelleway et al., 2017a). The millennial scale C_{org} accumulation rates estimated with ^{14}C in this study (2.0–14.8 g C_{org} m^{-2} yr^{-1}, Table 1) were also comparable to rates in other *Zostera* meadows (3.1–10.1 g C_{org} m^{-2} yr^{-1}) (Miyajima et al., 2015), *Enhalus* meadows (2.4–5.4 g C_{org} m^{-2} yr^{-1}) (Miyajima et al., 2015), and *P. australis* and *P. sinuosa* meadows (3.5–13.5 g C_{org} m^{-2} yr^{-1}) (Rozaimi et al., 2016; Serrano et al., 2016). However, the long-term C_{org} accumulation rate in *P. oceanica* meadows (a large species in the Mediterranean Sea) is 10-fold higher than the results in the present study (Mateo et al., 1997; Serrano et al., 2014). Our results support the proposal that the worldwide variability in seagrass carbon storage capability should be taken into account in the global estimate (Jankowska et al., 2016; Postlethwaite et al., 2018; Miyajima et al., 2015; Lavery et al., 2013).

References

Greiner, J. T., McGlathery, K. J., Gunnell, J., & McKee, B. A. (2013). Seagrass restoration enhances “blue carbon” sequestration in coastal waters. *PLOS ONE*, *8*, e72469.

Jankowska, E., Michel, L. N., Zaborska, A., & Włodarska-Kowalczuk, M. (2016). Sediment carbon sink in low-density temperate eelgrass meadows (Baltic Sea). *Journal of Geophysical Research: Biogeosciences*, *121*, 2918–2934.

Kelleway, J. J., Saintilan, N., Macreadie, P. I., Baldock, J. A., Heijnis, H., Zawadzki, A., Gadd, P., Jacobsen, G., & Ralph, P. J. (2017). Geochemical analyses reveal the importance of environmental history for blue carbon sequestration. *Journal of Geophysical Research: Biogeosciences*, *122*, 1789–1805.

Lavery, P. S., Mateo, M. Á., Serrano, O., & Rozaimi, M. (2013). Variability in the carbon storage of seagrass habitats and its implications for global estimates of blue carbon ecosystem service. *PLOS ONE*, *8*, e73748.

Mateo, M. Á., Romero, J., Pérez, M., Littler, M. M., & Littler, D. S. (1997). Dynamics of millenary organic deposits resulting from the growth of the Mediterranean seagrass *Posidonia oceanica*. *Estuarine, Coastal and Shelf Science*, *44*, 103–110.

Mazarrasa, I., Marbà, N., Garcia-Orellana, J., Masqué, P., Arias-Ortiz, A., & Duarte, C. M. (2017). Dynamics of carbon sources supporting burial in seagrass sediments under increasing anthropogenic pressure. *Limnology and Oceanography*, *62*, 1451–1465.

Miyajima, T., Hori, M., Hamaguchi, M., Shimabukuro, H., Adachi, H., Yamano, H., & Nakaoka, M. (2015). Geographic variability in organic carbon stock and accumulation rate in sediments of East and Southeast Asian seagrass meadows. *Global Biogeochemical Cycles*, *29*, 397–415.

Postlethwaite, V. R., McGowan, A. E., Kohfeld, K. E., Robinson, C. L. K., Pellatt, M. G. (2018). Low blue carbon storage in eelgrass (*Zostera marina*) meadows on the Pacific Coast of Canada. *PLOS ONE*, *13*, e0198348.

Rozaimi, M., Lavery, P. S., Serrano, O., & Kyrwood, D. (2016). Long-term carbon storage and its recent loss in an estuarine *Posidonia australis* meadow (Albany, Western Australia). *Estuarine, Coastal and Shelf Science*, *171*, 58–65.

Serrano, O., Lavery, P. S., Rozaimi, M., & Mateo, M. Á. (2014). Influence of water depth on the carbon sequestration capacity of seagrasses. *Global Biogeochemical Cycles*, *28*, 950–961.

Serrano, O., Ricart, A. M., Lavery, P. S., Mateo, M. Á., Arias-Ortiz, A., Masque, P., Rozaimi, M., Steven, A., & Duarte, C. M. (2016). Key biogeochemical factors affecting soil carbon storage in *Posidonia* meadows. *Biogeosciences*, *13*, 4581–4594.

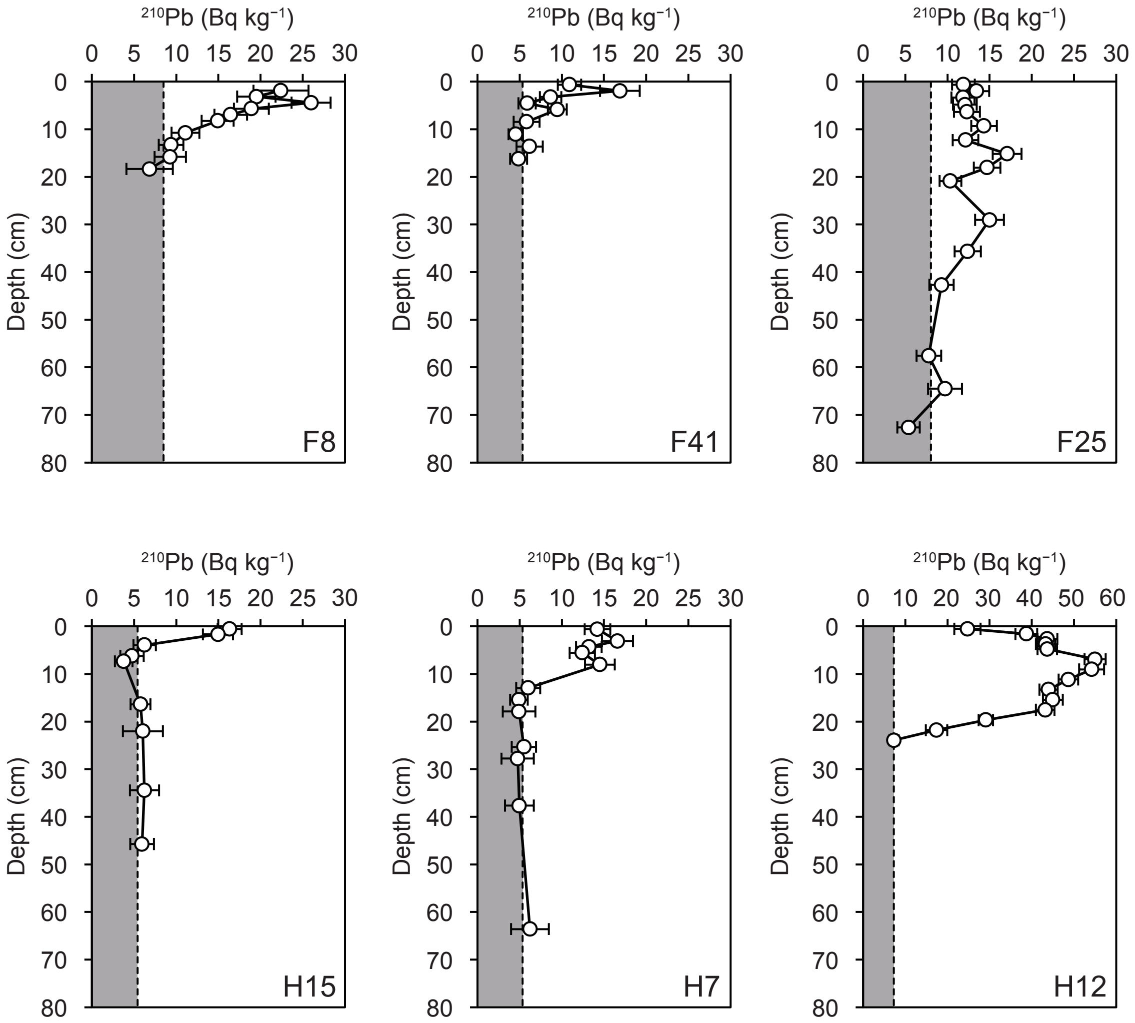

Figure S1. Measured depth profiles of ^{210}Pb concentration (±1σ). The grey shaded area indicates the supported ^{210}Pb ($^{210}Pb_{sup}$) in each core.

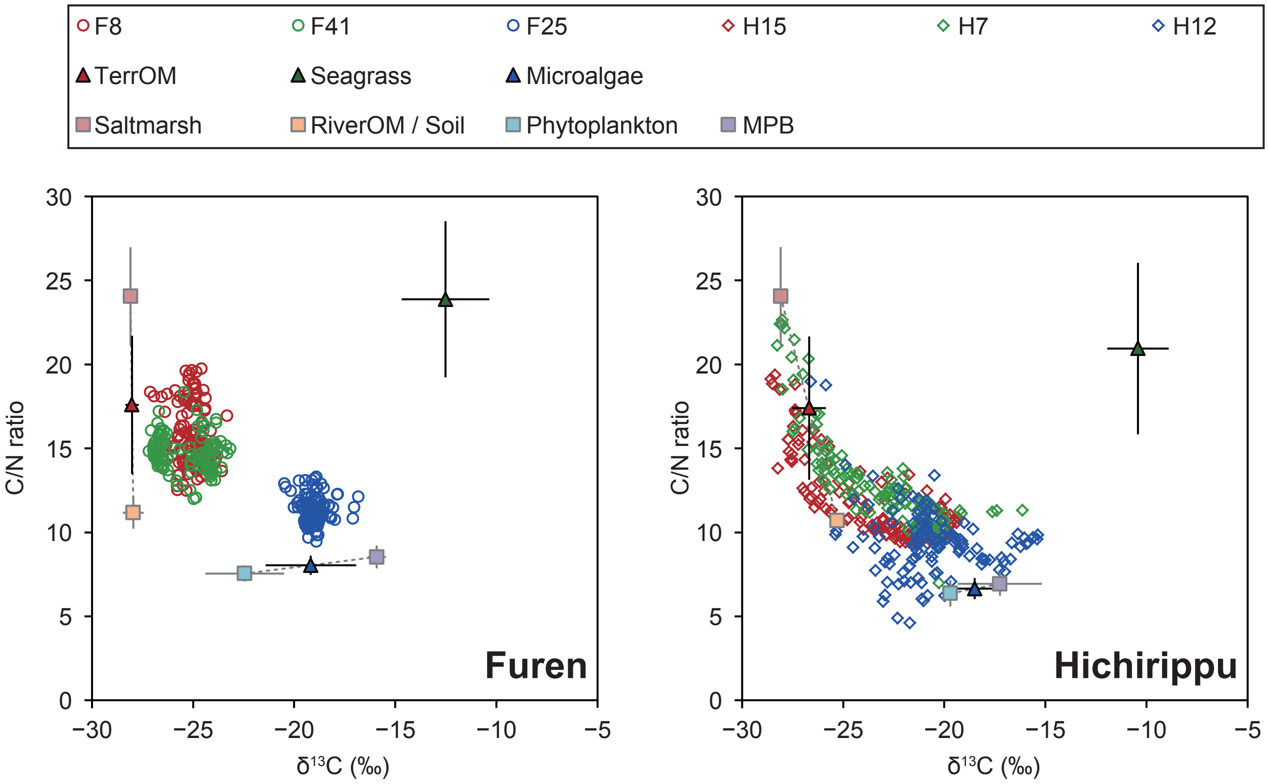

Figure S2. Isotopic and elemental signatures of C_{org} sources and sediment samples in Furen and Hichirippu lagoons. Error bars represent standard deviations of each source.

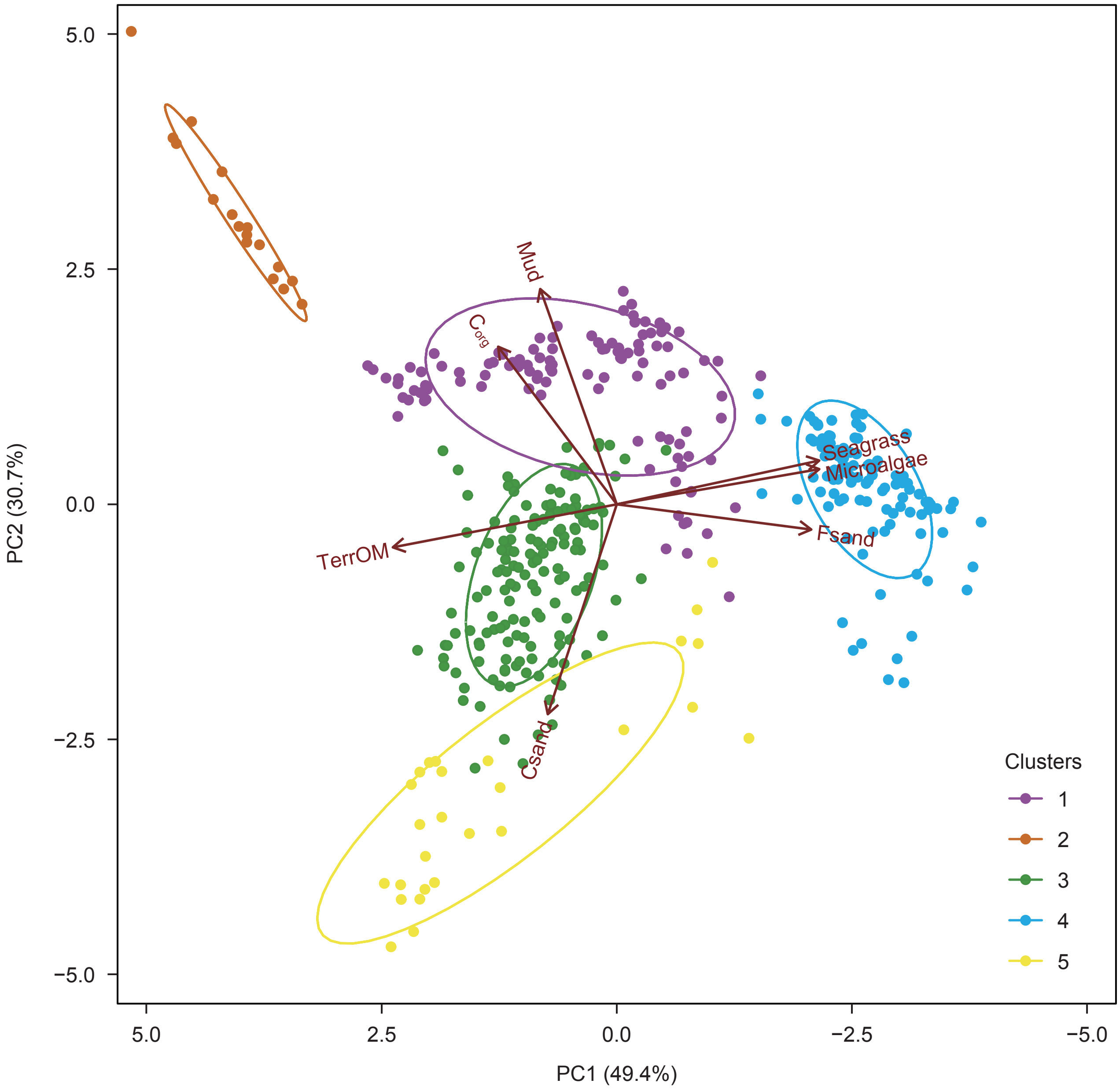

Figure S3. Principal component analysis of C_{org} concentration, grain-size composition (Csand, >500 µm; Fsand, 63–500 µm; Mud, <63 µm), and C_{org} source composition of the sediment samples identified by the change point analysis. The first two axes accounted for 80.1% of the variance. TerrOM, terrestrial-derived organic matter.
